# Aggressive juvenile ossifying fibroma of the ethmoid sinus with orbital and intracranial extension: A case report

**DOI:** 10.1016/j.ijscr.2022.107255

**Published:** 2022-06-01

**Authors:** Dalia Al Arfaj, Ali Almomen, Musab Bakri, Haifa Lafi Alenzi

**Affiliations:** a Department of Otorhinolaryngology Head and Neck Surgery, King Fahad Specialist Hospital, Dammam, Saudi Arabia; b Department of Medical Imaging, King Fahad Specialist Hospital, Dammam, Saudi Arabia; c Department of ENT, North Medical Tower, Arar, Saudi Arabia

**Keywords:** Ossifying fibroma, Juvenile ossifying fibroma, Fibroma, Orbital and intracranial extension, Nose, Paranasal sinuses, Case report

## Abstract

**Background:**

Juvenile ossifying fibroma is a rare benign destructive lesion of nose and paranasal sinuses. It occurs in the craniofacial bones of children below 15 years of age. It is usually discovered in the mandible and maxilla and rarely in the paranasal sinuses.

**Case presentation:**

We present a case of a 15-year-old girl with extensive right ethmoid sinus juvenile ossifying fibroma with intracranial and orbital involvement that was managed endoscopically at our center followed by lateral rhinotomy and frontal craniotomy with reconstruction due to the aggressive nature and recurrence of the disease. Patient was followed up post operatively for 3 years and was found free of symptoms ever since.

**Conclusion:**

In this case report we present our experience in managing this aggressive recurrent disease of juvenile ossifying fibroma which requires multiple endoscopic and open surgical procedures. Such pathology needs a close and long follow up due to the aggressive nature of this disease.

## Highlights

• Juvenile ossifying fibroma is a rare benign destructive lesion of nose and paranasal sinuses.
• Occurs in the craniofacial bones of children below 15 years. It is usually discovered in the mandible, maxilla and rarely in the paranasal sinuses.
• Most of the cases are asymptomatic and present lately when complications occur. Few cases involving the ethmoid sinus were reported.
• The main-stay management is debulking the lesion by preserving adjacent vital structures like the orbit.
• Long term follow-up is recommended due to high recurrence rate.

## 1 Introduction and importance

Ossifying fibroma (OF) is usually a benign lesion characterized by the replacement of normal bone by fibrous tissue containing varying amounts of mineralized material resembling bone [1]. It usually occurs in patients in their second to the fourth decade with a greater prevalence among females [2] and affects the mandible more than the maxilla, and rarely affects the paranasal sinuses [3], [4]. OF can, however, affect children under 15 years of age and hence came the name ‘juvenile ossifying fibroma’ (OSF) [5]. The latter is classified into two subtypes: Juvenile trabecular ossifying fibroma (JTOF) and Juvenile psammomatoid ossifying fibroma (JPOF).

JTOF usually occurs in the maxilla, and JPOF has an affinity to paranasal sinuses, and both are liable to recurrence [6]. Recurrence of JOF can be dealt with by surgical excision; however, aggressive JOF with a rapid growth rate needs to be treated with enbloc resection [7]. Kaban and his colleagues suggested a different protocol for the management of aggressive JOF. The protocol consisted of curettage or enucleation in combination with adjuvant interferon-alpha therapy for a year [8].

A previous meta-analysis was conducted in an attempt to obtain a comprehensive review of the literature published about JTOF and JPOF and help delineate the difference between the two. Patients with JPOF were found to be usually older than patients with JTOF. During treatment, the recurrence rate was higher in the case of JOF treatment by curettage and enucleation only and less when enucleation was followed by curettage/osteotomy [6].

The purpose of this study was to report a rare case of aggressive juvenile ossifying fibroma of the ethmoid sinus with orbital and intracranial extension and to review the published data on similar cases. The work has been reported in line with the SCARE criteria [23].

## 2 Case presentation

### 2.1 Juvenile active ossifying fibroma

A 15-year-old girl presented to King Fahad specialist hospital, Dammam, complaining of progressive right-sided eye proptosis associated with right-sided nasal obstruction. The proptosis developed gradually over the course of three months; however, the vision remained normal. Upon discovery, the patient was admitted, and pediatric, ophthalmology, otolaryngology, neurosurgery opinion was sought. When questioned, the patient denied experiencing any discomfort, headaches, changes in vision, or diplopia. There was no weight loss nor loss of appetite recorded. On inspection, no signs of allergic rhinitis or sinusitis were found.

After ocular examination, the patient showed right-sided eye proptosis, which is common in cases of ethmoidal involvement. For further examination, nasal endoscopy was performed. The endoscopy revealed several nasal polyps on the right side, as well as a deviated nasal septum to the left due to the strain created by the polyps.

### 2.2 CT scan

**Fig. 1:**
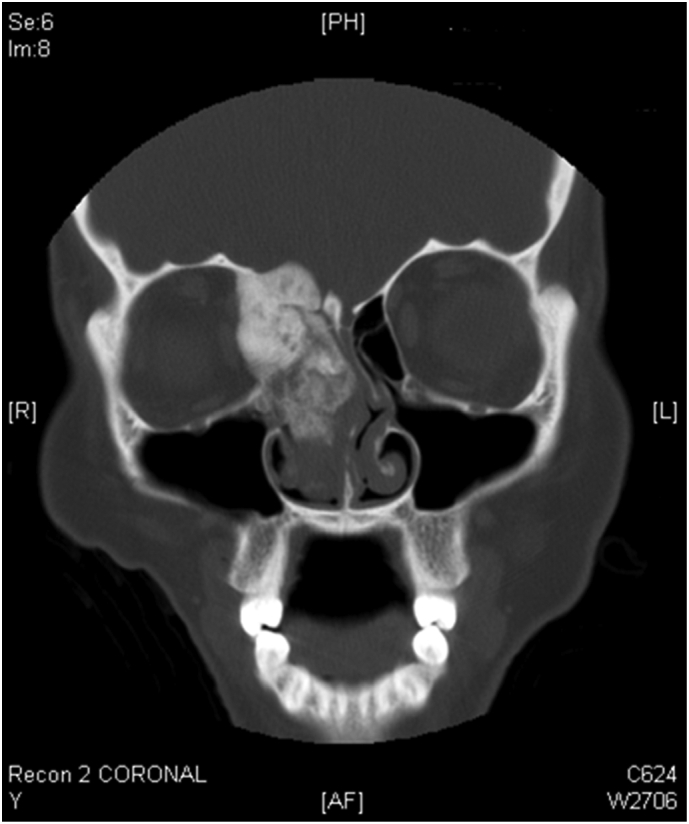
Paranasal coronal CT scan shows right ethmoid sinus and nasal cavity mass with orbital and intracranial involvement.

**Fig. 2:**
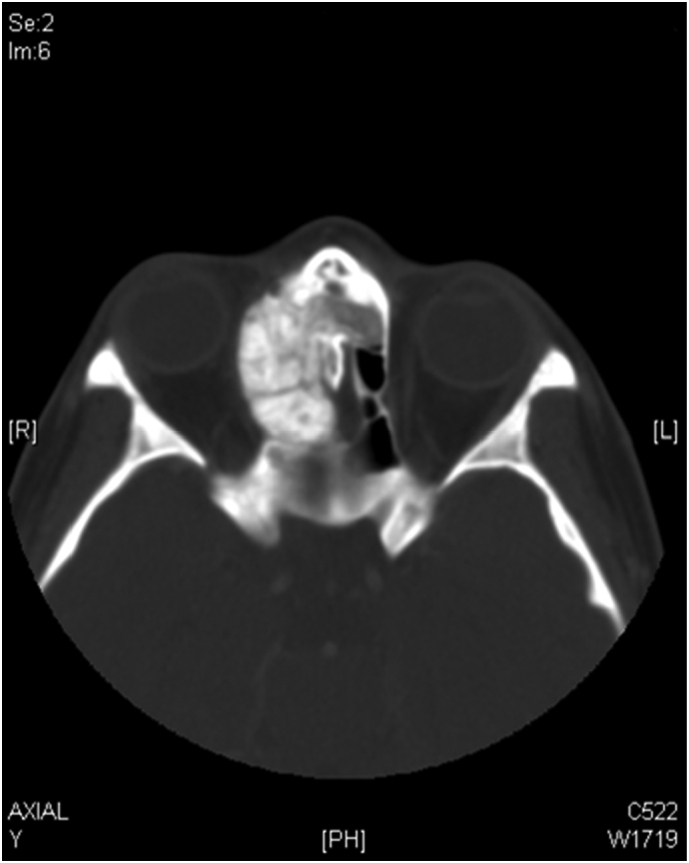
Paranasal axial CT scan shows right ethmoid sinus and nasal cavity mass with orbital and intracranial involvement.

A CT scan of the nose and paranasal sinuses revealed a large, calcified mass based on the right orbital plate; the mass is expansile with heterogeneous density and a ground-glass appearance, and it is correlated with the remodeling of the surrounding bony margins. With the obliteration of the right ostiomeatal complex, the mass has spread to the right ethmoid cavity. Orbital proptosis is present (Fig. 1, Fig. 2).

### 2.3 Surgical interference

**Fig. 3:**
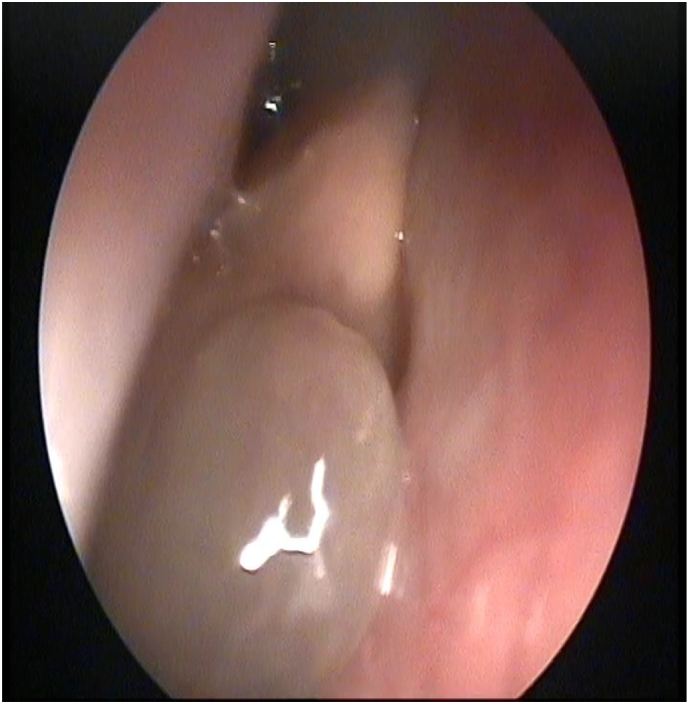
Intra-operative endoscopic image shows multiple nasal polyps.

**Fig. 4:**
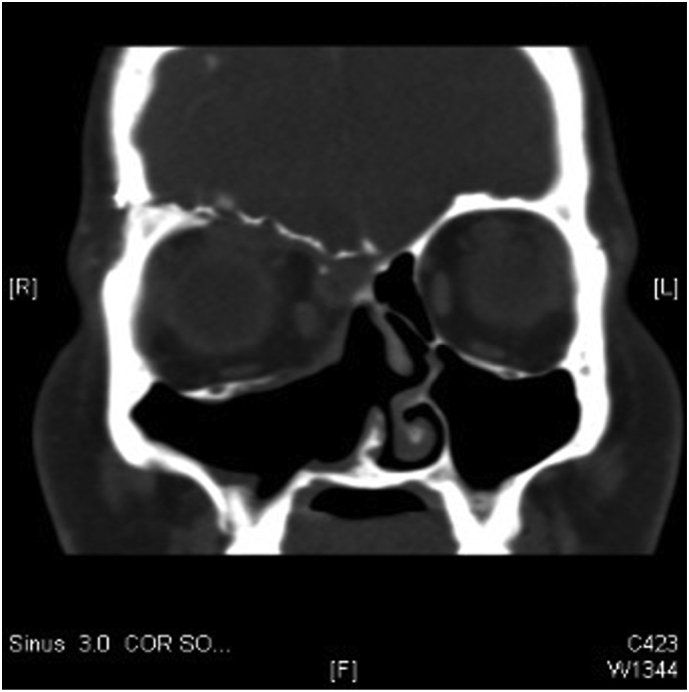
Post-operative paranasal coronal CT scan.

The patient underwent multiple operations, including endoscopic debulking of the lesion followed by lateral rhinotomy, and finally, frontal craniotomy with reconstruction as a combined procedure with the neurosurgery team (Fig. 3). Post-operative complications were noticed, which included temporary diplopia and mild enophthalmos. The patient was followed up in ENT/OPTHALOMOLGY/NEUROSURGERY clinic for three years with no evidence of recurrence clinically nor radiologically (Fig. 4). Her diplopia eventually resolved, and her vision improved.

## 3 Clinical discussion

In this report, we presented a case of aggressive juvenile active ossifying fibroma of the ethmoid sinus with orbital and intracranial extension. The case had to undergo multiple operations, including endoscopic debulking of the lesion followed by lateral rhinotomy, and finally, frontal craniotomy with reconstruction as a combined procedure with the neurosurgery team.

Surgical excision of the lesion is crucial because of its aggressive development and high rate of recurrence. Therefore, also, post-operative follow-up and inspection for recurrence are mandatory [9].

A previous case report of a psammomatoid ossifying fibroma (JPOF) in an 8-year-old boy reported recurrence within a year after treatment, even with surgical enucleation and curettage [10]. JPOF was also found to lead to secondary aneurysmal bone cysts of the maxilla with recurrency. A case report of a 10-year-old boy exhibited a recurrence within two years after surgical excision of a small swelling in the maxillary anterior region. The lesion was of massive size, extending anteriorly up to the right canine region, posteriorly up to the left side zygomatic bone, superiorly up to the left infraorbital margin, and inferiorly up to the alveolus [11].

JOF can also extend to the brain and the eye, as presented in our report. Another report with similar findings was of a 12-year-old boy who had a JPOF arising from paranasal sinuses and which in turn extended to the anterior skull base and the orbit. The diagnosis of similar cases, however, is a bit tricky given the similarity between them and other types of ossifying fibroma or meningioma [12], [13]. Another case report of a 13-year-old girl suffering from maxillary JTOF exhibited medial and lateral cortical expansion with extension to the floor of the orbit [14]. Involvement of the paranasal sinuses or the orbital bones may lead to the development of proptosis, as presented in our case and the previous case [14].

On radiological examination, lesions appear variable depending on the stage and the degree of calcification. JOF has three stages in radiographs; the first stage appears as a well-defined radiolucency without any sign of internal calcification, the second stage includes spots of radio-opaque material within the radiolucency. The third and the last stage is presented as a complete radio-opaque lesion [15]. Though the transformation into stage 3 may take years, in aggressive cases, it can take less than one year [16]. Our case appears to be in stage three.

In addition to this case, nasal polyps were also noted in previous cases of JOF, leading to nasal obstruction, which is a common symptom of JOF when it affects the sinuses [17], [18].

Despite its being benign, JOF tends to cause more serious damage and erosion, in addition to adherence to soft tissue in comparison with malignant lesions such as RMS or chondrosarcoma [19]. They both are usually confused with fibrous dysplasia (FD). Fibrous dysplasia is classified as a benign fibro-osseous lesion in which the normal bone is replaced with fibrous connective tissue due to a disturbance in bone metabolism. A key difference to differentiate the two is that OF has a well-defined edge which is not present in the case of FD. A case report of a patient with FD has meticulously examined the difference between the two. It was also mentioned that the cause behind FD might be a mutation that causes abnormal differentiation of osteoblasts and leads to the production of dysplastic bone [20], [21]. The etiology of OF is unclear but is thought to be related to multipotential mesenchymal cells of the periodontal ligament or to trauma [22].

## 4 Conclusion

Juvenile ossifying fibroma is a rare entity of benign fibro-osseous lesions. Most of the cases are asymptomatic and present lately when complications occur. Few cases involving the ethmoid sinus were reported. The main-stay management is debulking the lesion by preserving adjacent vital structures like the orbit. Long-term follow-up is recommended due to its high recurrence rate. In this report, we presented a case of aggressive juvenile ossifying fibroma of the ethmoid sinus with orbital and intracranial extension. The patient had no recurrence after surgical interference.

## Data availability

The data used to support the findings of this study are included within the article. Also, they are available from the corresponding author upon request.

## Consent

Written informed consent was obtained from the patient's next of kin for publication of this case report and accompanying images. A copy of the written consent is available for review by the Editor-in-Chief of this journal on request.

## Provenance and peer review

Not commissioned, externally peer-reviewed.

## Ethical approval

Ethical approval by IRB for case reports is not needed in this institution.

## Funding

This work was not supported by any forms of funding.

## Guarantor

Ali Almomen.

## Research registration number

This is not the first in man study.

## CRediT authorship contribution statement

Dalia Al Arfaj: data collection and analysis, writing the original manuscript draft.
Haifa Alenzi:, review and editing of the manuscript.
Musab Bakri: data analysis.
Ali Al Momen: study concept, data analysis, and final approval of manuscript.

## Declaration of competing interest

The authors declare that there is no conflict of interest regarding the publication of this paper.
